# Effectiveness of Hospital-directed Wellness Interventions in COVID-19: A Cross-sectional Survey

**DOI:** 10.5811/westjem.57306

**Published:** 2023-03-22

**Authors:** Adrian Cotarelo, Mary McLean, Nishad Rahman, Alina Mitina, Brenda Alves, Miriam Kulkarni

**Affiliations:** * St. John’s Riverside Hospital, Department of Emergency Medicine, Yonkers, New York; † Fire Department of the City of New York, FDNY Office of Medical Affairs, Brooklyn, New York; ‡ Northwell Health at North Shore/Long Island Jewish Medical Center, Department of Emergency Medicine, Long Island, New York; § AdventHealth Orlando, Department of Emergency Medicine, Orlando, Florida; ¶ United States Acute Care Solutions, Department of Clinical Innovation, Canton, Ohio; || Lake Erie College of Osteopathic Medicine, Erie, Pennsylvania; # LifeBridge Health, Department of Emergency Medicine, Baltimore, Maryland; ** Pace University College of Health Professions, Physician Assistant Studies Program, Pleasantville, New York

## Abstract

**Introduction:**

Hospitals have implemented various wellness interventions to offset the negative effects of coronavirus disease 2019 (COVID-19) on emergency physician morale and burnout. There is limited high quality evidence regarding effectiveness of hospital-directed wellness interventions, leaving hospitals without guidance on best practices. We sought to determine intervention effectiveness and frequency of use in the spring/summer 2020. The goal was to facilitate evidence-based guidance for hospital wellness program planning.

**Methods:**

This cross-sectional observational study we used a novel survey tool piloted at a single hospital and then distributed throughout the United States via major emergency medicine (EM) society listservs and closed social media groups. Subjects reported their morale levels using a slider scale from 1 (lowest) to 10 (highest) at the time of the survey and, retrospectively, at their respective COVID-19 peak in 2020. Subjects also rated effectiveness of wellness interventions using a Likert scale from 1 (not at all effective) to 5 (very effective). Subjects indicated their hospital’s usage frequency of common wellness interventions. We analyzed results using descriptive statistics and t-tests.

**Results:**

Of 76,100 EM society and closed social media group members, 522 (0.69%) subjects were enrolled. Study population demographics were similar to the national emergency physician population. Morale at the time of the survey was worse (mean [M] 4.36, SD 2.29) than the spring/summer 2020 peak (M 4.57, SD 2.13) [t(458)=−2.27, P=0.024]. The most effective interventions were hazard pay (M 3.59, SD 1.12), staff debriefing groups (M 3.51, SD 1.16), and free food (M 3.34, SD 1.14). The most frequently used interventions were free food (350/522, 67.1%), support sign display (300/522, 57.5%), and daily email updates (266/522, 51.0%). Infrequently used were hazard pay (53/522, 10.2%) and staff debriefing groups (127/522, 24.3%).

**Conclusion:**

There is discordance between the most effective and most frequently used hospital-directed wellness interventions. Only free food was both highly effective and frequently used. Hazard pay and staff debriefing groups were the two most effective interventions but were infrequently used. Daily email updates and support sign display were the most frequently used interventions but were not as effective. Hospitals should focus effort and resources on the most effective wellness interventions.

## INTRODUCTION

### Background

Burnout was already an issue for half of United States (US) emergency physicians (EP) in the years leading up to the coronavirus disease 2019 (COVID-19) pandemic.^1^–^6^ A national survey conducted from 2011–2014 revealed that physicians in frontline specialties are at greatest risk of burnout,^7^ and a 2018 review suggested that healthcare organizations already had insufficient basic resources to support physician wellness.^8^ Attempts have been made to ameliorate this concerning trend. Particularly among emergency medicine (EM) residency programs, over 162 unique wellness interventions have been described. The most commonly addressed themes of these interventions were program factors such as culture; environmental and clinical factors; and wellness activities, practices, and resources.^9^ Despite the implementation of these numerous interventions, a review study found that prior to the COVID-19 pandemic there has been little high mquality literature assessing the effectiveness of wellness interventions targeting EM residents.^10^

During the 2020 COVID-19 surges, EPs reported increased work-related anxiety, emotional exhaustion, and burnout.^11^ Despite these reports, the EP burnout rate showed a modest increase from 43% in 2019^6^ to 44% in the fall 2020.^12^ However, the issue of burnout has worsened markedly since then, even as the initial COVID-19 surges have waned.^13^–^14^ Among EM residents who worked during surges, 35% experienced acute post-traumatic symptoms.^15^ Common causes have been found to center around the themes of moral distress regarding patient deaths, resource allocation/scarcity, personal safety, economic insecurity, social/family life disruption, stigmatization of healthcare workers, and a sense of powerlessness.^16^

Recent COVID-19 pandemic-era literature has discussed how best to mitigate this issue. Some have recommended taking steps to improve healthcare workers’ exercise, food, and diet practices,^17^ but as Li-Sauerwine et al discuss,^9^ these recommendations are limited to personal factors as indicated by the National Academy of Medicine Model of Clinician Well-Being and Resilience.^8^ Specifically among EM residency programs, many have implemented additional wellness interventions beyond the minimum requirements of the Accreditation Council for Graduate Medical Education (ACGME),^18^ such as obtaining outside food donations, holding virtual social gatherings, and establishing new wellness/respite spaces.^19^ However, lack of high quality evidence on intervention effectiveness leaves hospitals and residency programs to guess which methods will work.

One study conducted in November 2020 revealed that several themes increased feelings of joy and fulfillment for frontline healthcare workers, including meaningful practitioner-patient interactions, team camaraderie, teaching/mentoring, physical activity, and time with family/friends.^20^ Thus far in the COVID-19 pandemic, the best evidence-based recommendations for hospital wellness interventions have been to focus on the following resources: social, leadership, financial, and mental health support; meeting safety needs; and providing childcare options.^21^ One specific intervention—a facilitated physician peer-support group model—was piloted across 10 hospitals and showed promise in improving anxiety, depression, distress, and burnout.^22^ However, no study has asked participants to rate the effectiveness of hospital wellness interventions during the COVID-19 pandemic era.

**Population Health Research Capsule**

What do we already know about this issue?

*Hospitals implemented wellness interventions to offset the effects of COVID-19 on physician morale, but there is little evidence-based guidance on their effectiveness*.

What was the research question?

*What is the perceived effectiveness of hospital-directed wellness interventions on emergency physicians’ morale?*

What was the major finding of the study?

*Hazard pay, debriefing groups, and free food were the most effective interventions. Of these three, only free food was frequently implemented*.

How does this improve population health?

*This study provides guidance for hospitals to refocus their wellness planning efforts to use the most effective interventions*.

## OBJECTIVES

We aimed to assess the effectiveness and use of hospital-directed wellness interventions from the perspective of EPs in the first surges of the COVID-19 pandemic in the US. The goal was to provide evidence-based recommendations for future hospital wellness plans both during and after COVID-19 surges. The hypotheses were that some hospital-directed wellness interventions are significantly more effective to subjects’ personal well-being than others; that some highly effective interventions are infrequently used; and that some ineffective interventions are frequently used.

## METHODS

### Human Subjects

This study was approved as exempt by the institutional review board. Study procedures were disclosed to subjects prior to answering an informed consent question at the beginning of the survey. The survey was anonymous.

### Study Setting and Population

This cross-sectional survey used convenience sampling in a virtual setting. No incentives were offered. Those who took the survey included EP attendings, fellows, and residents practicing or training in the US and outlying territories. Included were subjects who completed the “Information, Consent, and Demographics” page of the survey *and* answered at least one question in the “Wellness Initiatives” page. We analyzed the subjects’ data only for the questions the participants answered. We excluded subjects who completed only the “Information, Consent, and Demographics” section.

### Survey Development and Pilot Testing

Survey content was developed by author team consensus, with additional guidance from a townhall-style discussion with 25 attending and resident EPs at the primary institution on May 20, 2020. All attendees at this townhall had the lived experience of practicing medicine in the emergency department and/or intensive care unit during spring 2020 in Westchester County, NY, which was the second hardest hit county in New York State as of July 1, 2020, based on COVID-19 case numbers per capita.^23^ Two senior authors were also members of regional groups of academic institutions that had met and discussed EP wellness challenges and hospital responses.

See Appendix A for the recruitment script. See Appendix B for the complete survey tool including informed consent. In addition to hospital demographic information, we included questions about hospital-directed wellness interventions. Subjects’ reports of intervention effectiveness on their own personal wellness were assessed using a Likert scale from 1 (not at all effective) to 5 (very effective). We assessed the subjects’ reports of their personal morale levels using a slider scale from 1 (lowest) to 10 (highest) at time of survey and, retrospectively, at the first US COVID-19 surge peak in spring/summer 2020. Subjects were also given the opportunity to contribute free-text comments on wellness interventions they wish had been offered, other things that may have improved morale, and additional suggestions or comments. We collected this free-text data for the purpose of future thematic analysis (a planned future direction for this research group), but this data was not employed in the present study.

The survey was sent to a pilot group of resident and attending physicians at a single hospital for clarity and usability feedback, and for preliminary analysis, prior to national-scale distribution. No clarity or usability issues were cited, and no changes to the survey instrument were required prior to national distribution.

### Study Protocol and Statistical Analysis

We used the electronic platform SurveyMonkey (SurveyMonkey Enterprise, San Mateo, CA) to construct and distribute the survey. In deciding on survey distribution methods, we aimed to reach the largest and most diverse group of EPs possible. To achieve this aim, we used listservs associated with The American College of Emergency Physicians (ACEP), the Council of Residency Directors in Emergency Medicine (CORD), and the Society for Academic Emergency Medicine (SAEM), and posts on two closed Facebook social media groups EMDocs and Emergency Physician Forum. These organizations and groups had a collective membership of 76,100 members at the time of data collection from July 25–August 9, 2020. (See Appendix C for medical society listserv and closed Facebook group membership numbers at the time of survey distribution and active data collection.).

Recruitment occurred online via listserv email invitations and closed social media group posts, including ACEP, CORD, SAEM, and the closed Facebook groups EMDocs and Emergency Physician Forum. We determined these platforms to be the most accessible for the wider population of EPs. On average, two contacts were attempted on each of these five platforms. We analyzed data with descriptive statistics and paired *t*-tests using R version 3.6.1 for Windows (R Foundation for Statistical Computing, Vienna, Austria). In reporting results of the study, we used the recommendations outlined by STROBE (Strengthening the Reporting of Observational studies in Epidemiology). See Appendix D for the STROBE checklist used.

## RESULTS

### Pilot Data

Of 16 pilot subjects who completed the survey from June 29–July 10, 2020, two (12.5%) were attending physicians and 14 (87.5%) were resident physicians. Preliminary analyses of pilot data showed the most effective interventions to be hazard pay (mean [M] 4.5, SD 0.78), free food at work (M 4.2, SD 0.97), and staff debriefing groups (M 3.4, SD 1.3). Also, morale was reported to be lower at the time of the pilot survey (M 3.8, SD 2.3) than during the first peak (M 5.1, SD 2.3). The pilot population and main population demographics were dissimilar in terms of practice location and breakdown of participant level of training. However, the major study outcomes of personal morale and perceived intervention effectiveness were found to follow the same patterns. Thus, we incorporated the pilot data into the main analysis.

### Enrollment and Demographics

**Figure 1:**
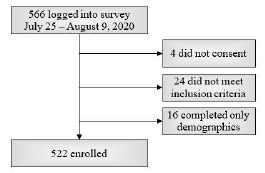
Enrollment flowsheet. Included were consenting EP attendings, fellows, and residents currently practicing or training in the US and outlying territories who answered questions in both the “Demographics” and “Wellness Initiatives” sections of the survey.

**Figure 2:**
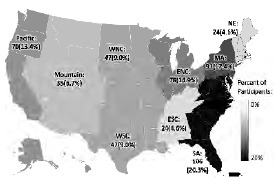
Subjects by United States region. Regional breakdown based on prior emergency physician workforce studies. Puerto Rico was included in the South Atlantic region. *ENC*, East North Central; *ESC*, East South Central; *MA*, Mid Atlantic; *NE*, Northeast; *SA*, South Atlantic; *WNC*, West North Central; *WSC*, West South Central.

A total of 566 subjects logged into the survey, and 522 subjects were enrolled. The barriers to calculating a response rate are discussed in the “Limitations” section of this paper. The enrollment flowsheet is shown in Figure 1. Study group demographic characteristics are shown in Table 1. Participation by US region is depicted in Figure 2.

### Main Results

**Figure 3:**
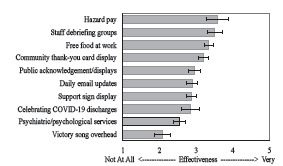
Ranking of hospital-directed wellness intervention effectiveness on a Likert scale from 1 (not at all effective) to 5 (very effective). Error bars represent the 95% confidence interval. *COVID-19*, coronavirus disease 2019.

Morale at the time of the survey (M 4.36, SD 2.29) was significantly worse than morale during the initial spring/summer surge (M 4.57, SD 2.13); [t(458)=−2.27, *P*=0.02). See Table 2 for frequency and effectiveness of hospital wellness interventions. See Figure 3 for the ranking of hospital wellness interventions based on participant reports of effectiveness on their personal wellness. See Table 3 for analyses of hazard pay amounts, the details of which are provided because hazard pay was ranked as the most effective hospital-directed wellness intervention.

## DISCUSSION

### Effectiveness of Interventions

The most effective intervention was found to be hospital-sponsored hazard payment. This was also one of the least frequently used interventions. It appears to be a relatively novel intervention, and we were unable to find any previous research regarding the effectiveness of such hospital-sponsored hazard pay. Subjects’ reported hazard payment amounts ranged remarkably. It is interesting to note that 48% of the subjects who reported a non-zero payment and answered the hazard pay sufficiency question felt the amount was sufficient. Attending and fellow physicians found hazard pay amounts to be sufficient more frequently than resident physicians, but the mean hazard pay reported by attendings and fellows was higher than for residents.

Notably, we found that staff debriefing groups were also a highly effective intervention, although this was only reported by 25% of the subjects. This is a low-cost intervention that could be quickly implemented and should be within the capacity of every hospital. This is consistent with the work of Schneider and Weigi, who found that peer support and pay were associated with improved practitioner well-being,^25^ and with other studies that have found peer support groups to be effective in supporting practitioner wellness.^26^ The results of this study support the use of peer support groups to promote wellness during pandemics or other times of stress. The only other interventions that had greater-than-average effectiveness ratings were free food at work and “thank you” cards.

The most frequent interventions were free food, support sign display, and daily email updates. These interventions may require very few hospital resources to accomplish. For example, it is possible that free food at work may have been subsidized by numerous different sources, including the hospital itself or by local community members or businesses that wished to show appreciation. It is important to acknowledge that any food provided by the hospital itself (not by the community in the outpouring of support during the surges) may have had different effects on physician wellness; however, this was not something the survey tool assessed.

Among the least effective interventions was the presence of psychiatric/psychological support services. Only 36% of subjects reported having psychiatric or counseling services made available to them by their hospital. This was surprising because providing counseling services is frequently recommended to improve physician wellness after exposure to stressful or traumatic events. In fact, residency programs are required by the ACGME to make counseling on demand available to their residents.^18^ Also among the least effective interventions was the practice of playing a “victory” song overhead in the hospital for COVID-19 patient successes, purported to boost morale. We had a particular interest in this intervention because of the potential for overhead “victory” songs to interrupt conversations at inopportune times (eg, during delivery of bad news to loved ones of patients).

### External Validity

The survey instrument content was not based on any prior validated assessment but was developed by consensus of EP residents and attendings with the lived experience of working during a significant COVID-19 surge and was piloted prior to national distribution. The survey items were carefully constructed and closely related to the research questions.

In terms of population validity, while we do believe that using large medical societies and closed social media groups as key recruitment platforms maximized inclusivity, this brings external validity into question because not all EPs are members of medical societies or closed social media groups. The study sample was, however, largely representative of the overall US EP population in terms of the demographic characteristics collected. At the time of data collection, there were 8,642 EM residents^27^ and 48,835 active EPs in the US.^24^ The regional distribution and urban rural distribution of subjects adequately mirrors the demographics of the EM workforce as described in Table 4. No information on breakdown of greater population hospital type was available.

Lastly, we used a specific disaster, the COVID-19 pandemic, to measure EP personal morale and perceived effectiveness of hospital-directed wellness interventions. However, we do believe our findings have transferability to other contexts such as future epidemics, pandemics, natural disasters, and other situations that increase stressors on EPs and put them at higher risk of burnout and low morale.

### Big-picture Meaning

Trends in the burnout rates for EPs can be followed over the past decade from large annual survey reports. The reports reveal two peaks in EP burnout, both closely trailing epidemic/pandemic scares in the US. The first peak was in 2016, two years after the Ebola virus was detected in the US. (EP burnout rates were 52% in 2014,^1^ 55% in 2015,^2^ and 59% in 2016^3^ – an all-time high for EM at the time – before dropping to 45% in 2017^4^). The trend following the COVID-19 pandemic has mirrored the trend following the Ebola epidemic, with EP burnout rates at 44% in 2020,^12^ 60% in 2021,^13^ and 65% in 2022.^14^ This 2022 figure is another all-time high, and 2022 is the second year in a row that EPs have experienced the highest burnout rate of all medical specialties.

Although the US case positivity rate was drastically different between Ebola and COVID-19, we do speculate that the Ebola scare was a significant factor in the 2015 and 2016 burnout rates. Even with low case positivity rates in the US, the Ebola epidemic took a psychological toll on healthcare workers.^29^ The reason for the delayed rise in burnout after these pandemic/epidemic scares is likely multifactorial. The American Psychological Association (APA) argues that although longer work hours and home demands have been commonplace since the first surges, these stressors have now become persistent and indefinite, and exposure to such chronic states of stress increases the risk of burnout.^30^ The APA also cites public resistance to COVID-19 prevention measures as another potential persistent stressor that may affect frontline workers in particular.^30^

Although our subjects were surveyed at varied times relative to their local COVID-19 surge, the majority did report that their morale at the time of the survey was worse than during their respective surge peak. This information, and the annual EP survey of burnout trends, not only suggests that there is a correlation between low morale/high burnout and epidemic/pandemic scares, but also that the detrimental psychological effects on physicians last long after disease incidence wanes. While overall hospital admission rates for COVID-19 are significantly lower than peak rates,^31^ the pandemic has been ongoing now for more than two years, with unprecedented effects on clinician wellness. Our study shows that common hospital-directed wellness interventions vary greatly in effectiveness, and continued research is necessary to identify targeted interventions that can assist hospitals in supporting their EPs as the pandemic continues, even as COVID-19 rates continue to decrease in the US.

## LIMITATIONS

The survey was an original and unvalidated tool. Although it was piloted at a single, suburban community hospital to glean preliminary evidence of response process validity and improve the survey prior to national distribution, the pilot group was not representative of the main study population in terms of attending/resident breakdown. However, data trends for main outcomes were similar between the pilot and main study populations. One limitation in survey tool clarity that was not brought up in the pilot is that subjects may have had different definitions of “informal staff-debriefing groups,” which may have impacted the reported frequency and effectiveness of this intervention.

Subjects were surveyed during a discrete two-week timeframe, but surges at their hospitals peaked on various dates. Thus, there was potential for varied degrees of recall bias, particularly regarding retrospective morale levels. The effectiveness of interventions may also vary based on their timing relative to peaks and valleys in COVID-19 incidence; this was not possible to measure with the methodology used.

Study enrollment was voluntary (specifically, a voluntary response sampling method was used), and thus the survey results were vulnerable to self-selection bias (ie, some participants were inherently more likely to volunteer). In using this method, subjects with strong feelings about hospital-directed wellness interventions may have been more likely to participate, and subjects with neutral feelings may have been unintentionally excluded. We believe this source of bias would have increased the variability of reports of morale and intervention effectiveness (stronger subject opinions on both sides). Similarly, non-response bias may also have resulted from failure to enroll potential subjects who had experienced the peak of their local surge closer to the two-week survey administration period and were working longer hours or otherwise preoccupied. Given the trend in reported morale levels, the potential exclusion of these subjects may have biased our sample toward increased changes in pre/post-surge morale reports, as well as decreased absolute morale levels at the time of survey administration. Our sample also included a heterogeneous group of attendings, fellows, and residents with different wellness needs.

We were unable to precisely calculate response rate. Medical society members do not necessarily check organizational message boards, and may ignore, delete, or opt out of listserv emails. The rate of dual membership in these medical societies and groups could not be determined, thereby further limiting our response rate calculation; however, we acknowledge that dual membership is common and, thus, we speculate that we reached out to far fewer than 76,100 potential subjects. To mitigate this issue, future studies will use a more structured method of direct email contact and open rate tracking to obtain response-rate denominators. Further research will also employ thematic analysis of the free-text commentary provided by subjects in this survey, the results for which were extensive but outside the scope of the present study. Further research is also necessary to directly measure the effect of these interventions on burnout (rather than subjective effectiveness ratings).

## CONCLUSION

There is discordance between the most effective and most frequently used hospital-directed wellness interventions. Only free food was both highly effective and frequently used. Hazard pay and staff debriefing groups were the two most effective interventions but were infrequently used. Daily email updates and support sign display were the most frequently used interventions but were not as effective. Hospitals should consider the relative effectiveness of these wellness interventions when deciding where to focus their efforts and resources.

## Supplementary Information



## Figures and Tables

**Table 1:** Study population demographic characteristics. “Other” hospital types include military, Veterans Administration, and all other reported types.

Measure	n (%)
Level of training	
Attending	436 (83.52%)
Fellow	16 (3.07%)
Resident	70 (13.41%)
Hospital setting	
Urban	279 (53.45%)
Suburban	171 (32.76%)
Rural	64 (12.26%)
Other	8 (1.53%)
Hospital type	
Academic/university	213 (40.80%)
Community	268 (51.34%)
County	22 (4.21%)
Other	19 (3.63%)

**Table 2:** Descriptive data on frequency and effectiveness of hospital-directed COVID-19 wellness interventions, as reported by emergency physicians in the United States.

Intervention	Frequency (%)	Effectiveness (1–5)	

		Median	Mean (SD)
Hazard pay	53 (10.2%)	4	3.59 (1.12)
Staff debriefing groups	127 (24.3%)	4	3.51 (1.16)
Free food at work	350 (67.1%)	3	3.34 (1.14)
Community “thank you” card display	254 (48.7%)	3	3.21 (1.11)
Public acknowledgment/displays*	231 (44.3%)	3	2.96 (1.24)
Daily email updates†	266 (51.0%)	3	2.90 (1.25)
Support sign display	300 (57.5%)	3	2.87 (1.14)
Celebrating COVID-19 discharges	92 (17.7%)	3	2.85 (1.23)
Psychiatric/psychological services	188 (36.0%)	3	2.55 (1.12)
“Victory” song overhead	100 (19.2%)	2	2.09 (1.12)
Other support	17 (3.26%)	N/A	N/A
No support	23 (4.41%)	N/A	N/A

* “Public acknowledgment/displays” includes applause for hospital staff, military jets overhead, emergency medical services/fire department/police display of lights/sirens, etc.

† “Daily email updates” are emails to employees by hospital administration or other staff.

*COVID-19*, coronavirus disease 2019.

**Table 4:** Study population vs emergency physician workforce demographics.

Demographic characteristic	Study population (%)	EP workforce (%)
Level of training*		
Attending (Including Fellows)	87	85
Resident	13	15
Region†		
New England	5	6
Mid Atlantic	17	12
East North Central	15	15
West North Central	9	6
South Atlantic	20	20
East South Central	5	5
West South Central	9	11
Mountain	7	9
Pacific	13	17
Geographic Setting‡		
Urban	86	92
Rural	14	8

* Greater population level of training was based on 2020 Accreditation Council for Graduate Medical Education reports.27

† Greater population region was based on the 2020 Workforce report.24

‡ Greater population geographic setting was based on the 2020 Workforce report,24 which uses only urban or rural categories, consistent with modern Urban Influence Codes.28 Subjects reporting suburban setting are categorized as urban.

*EP*, emergency physician.
