# Supplementary material for: Effectiveness of Hospital-directed Wellness Interventions in COVID-19: A Cross-sectional Survey
Source: West J Emerg Med. 2023 Mar 22;24(3):597–604. doi: 10.5811/westjem.57306 (PMC10284535; doi:10.5811/westjem.57306)
Supplement: Supplementary file 1 [file wjem-24-597-s001.docx]

**APPENDICES**

**Appendix A: Recruitment Script**

*Subject Line: Impact of COVID-19 Wellness Initiatives*

Dear Colleagues,

The COVID-19 pandemic had a profound impact, both physical and emotional, on every member of the healthcare team. Institutions implemented various initiatives to support wellness during this difficult time. Now more than ever, it is crucial that we evaluate the effect of these initiatives on our healthcare community.

Emergency physicians, please help by taking this important 6-minute survey on COVID-19 wellness interventions. It is voluntary, anonymous, and can be taken on a smartphone, tablet, or computer. We will use the answers to assess and develop recommendations to improve wellness initiatives for high-stress events, such as current and future waves of the pandemic.

Hyperlink <Survey: Impact of Wellness Initiatives During the COVID-19 Pandemic>

This survey is a part of an IRB approved nationwide research project assessing the perceived efficacy of hospital wellness interventions for physicians during the COVID-19 pandemic. Please reach out if you have questions or concerns regarding this survey or the study itself. The primary site IRB contact email is SJRH.IRB@gmail.com

Gratefully,

The COVID-19 Wellness Initiative Team

St. John's Riverside Hospital

967 North Broadway

Yonkers, NY 10701

**Appendix B: Survey Tool**

*Survey Title: Impact of Wellness Initiatives During the COVID-19 Pandemic*

Page 1 - Information, Consent, and Demographics

Dear Respondents,

The COVID-19 pandemic had a profound impact, both physical and emotional, on every member of the healthcare team. Institutions implemented various initiatives to support wellness during this difficult time. Now more than ever, it is crucial that we evaluate the effect of these initiatives on our healthcare community.

Emergency and Critical Care Physicians, please help by taking this important 6-minute survey on COVID-19 wellness interventions. It is voluntary, anonymous, and can be taken on a smartphone, tablet, or computer. We will use the answers to assess and develop recommendations to improve wellness initiatives for high-stress events, such as current and future waves of this pandemic.

This survey is a part of an IRB approved nationwide research project assessing the perceived efficacy of hospital wellness interventions for physicians during the COVID-19 pandemic. Please reach out if you have questions or concerns regarding this survey or the study itself. The primary site IRB contact email is SJRH.IRB@gmail.com

Top of Form

1. Do you consent to have your survey answers entered into the study? (Select one)

- Yes
- No

2. What type of facility do you primarily work at? (Select one)

- Academic Hospital
- Community Hospital
- County Hospital
- Military Hospital
- Other (specify)

3. Which of the following best describes your practice setting? (Select one)

- Rural
- Urban
- Suburban
- Other (specify)

4. What role(s) do you hold at your facility? (Select all that apply)

- Attending Physician
- Fellow Physician
- Resident Physician
- Other (specify)

5. What is your medical specialty? (Select all that apply)

- Emergency Medicine
- Critical Care
- Pulmonology
- Internal Medicine
- Other (specify)

6. In which hospital area(s) did you work most in the COVID pandemic? (Select all that apply)

- Emergency Department
- Critical Care/ICU
- Internal Medicine/Floors
- Non-patient care role
- Did not work during the pandemic
- Other (specify)

7. During what period of the COVID pandemic did you primarily work? (Select all that apply)

- Before Peak
- Peak
- After Peak
- Did not work during the pandemic
- Other (specify)

8. At what time of the day did you primarily work? (Select one)


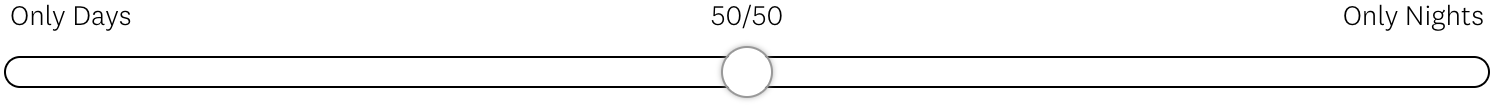


9. In what state did you primarily work during the COVID-19 pandemic? (Select one or specify)


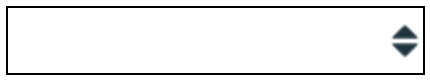


Page 2: Wellness Initiatives

Given the high-stress environment in hospitals during the COVID-19 pandemic, facilities across the country have launched wellness initiatives in the hopes of supporting their employees as much as possible. These include direct payments or hazard pay, daily email updates, free food at work, psychiatric services, public celebration of successful discharges, support signs within and around the hospital, thank-you cards from the community, and public acknowledgment such as flyovers, parking lot dances or displays, and public clapping/applause for hospital staff.

Additionally, many hospitals have adopted a “Victory Song”, where a short song is played through the hospital overhead system signaling either the successful discharge or extubation of a COVID-19 patient. This system is intended to provide hope and support to hospital employees, who are able to identify a positive patient outcome that they may not have personally witnessed.

10. How do you characterize your morale *at this moment* as a result of the COVID pandemic?


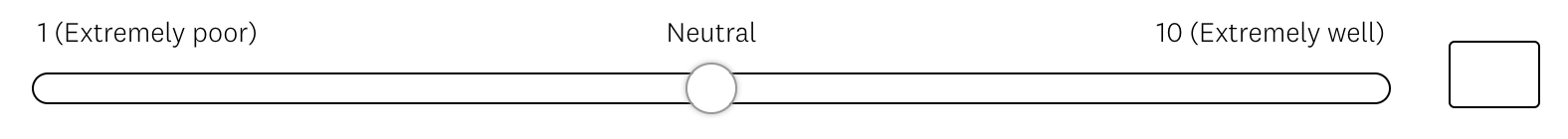


11. How do you characterize your morale *during the peak period* of the COVID pandemic?


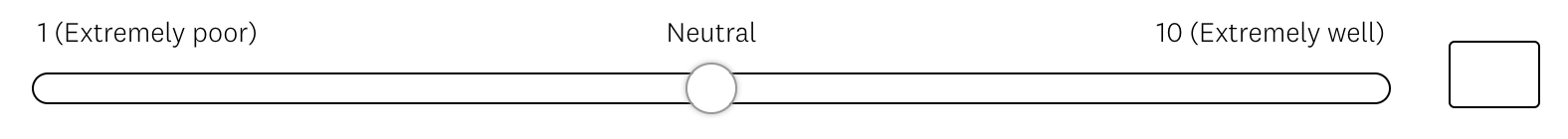


12. Which of these wellness initiatives were attempted at your facility? (Select all that apply)

- Victory Song playing overhead
- Direct Payment/Hazard Pay
- Psychiatric/Psychological services
- Free food at work, including from the community
- Public celebration of successful COVID-19 discharges
- Informal debriefing sessions among staff
- Displaying support signs
- Display of thank-you cards from the community
- Public acknowledgement/displays (e.g., applause for hospital staff, military jets overhead, EMS/FD/PD display of lights/sirens, etc.)
- Daily email updates from hospital administration or other staff
- None of the above
- Other (specify)

13. When does your facility play a Victory Song? (Select all that apply)

- When a COVID-19 patient is successfully discharged from the hospital
- When a COVID-19 patient is extubated successfully
- Periodically throughout the day regardless of COVID-19 clinical events
- Never
- Other (specify)

14. When your institution plays a Victory Song, where does it play? (Select all that apply)

- Floors
- ICU
- Emergency Department
- All other areas
- N/A
- Other (specify)

15. Have you had any of the following experiences with the Victory Song? (Check all that apply)

- Victory Song playing during a cardiac arrest or patient code.
- Victory Song playing while giving bad news to a family/friend or patient.
- Victory Song playing shortly after a patient death.
- Patient or family/friend became upset by the victory song playing
- Victory song played while discussing code status with patient and/or family/friend
- None
- N/A
- Other (specify)

16. Overall, how effective were the following wellness initiatives? Rate the effectiveness on a scale of 1 (not at all effective) to 5 (very effective). *Public acknowledgement/displays* includes applause for hospital staff, military jets overhead, EMS/FD/PD display of lights/sirens, etc.

*Daily email updates* include update emails from hospital administration or other staff.

| Initiative | 1 | 2 | 3 | 4 | 5 | N/A |
| --- | --- | --- | --- | --- | --- | --- |
| Victory Song playing overhead | Ο | Ο | Ο | Ο | Ο | Ο |
| Direct Payment/Hazard Pay | Ο | Ο | Ο | Ο | Ο | Ο |
| Psychiatric/Psychological services | Ο | Ο | Ο | Ο | Ο | Ο |
| Free food at work, including from the community | Ο | Ο | Ο | Ο | Ο | Ο |
| Public celebration of successful COVID discharges | Ο | Ο | Ο | Ο | Ο | Ο |
| Informal debriefing sessions among staff | Ο | Ο | Ο | Ο | Ο | Ο |
| Displaying support signs | Ο | Ο | Ο | Ο | Ο | Ο |
| Display of thank-you cards from the community | Ο | Ο | Ο | Ο | Ο | Ο |
| Public acknowledgement/displays* | Ο | Ο | Ο | Ο | Ο | Ο |
| Daily email updates** | Ο | Ο | Ο | Ο | Ο | Ο |
| None of the above | Ο | Ο | Ο | Ο | Ο | Ο |
| Other (specify) | Ο | Ο | Ο | Ο | Ο | Ο |

17. How much hazard pay/direct payment did you receive from your institution (total)?


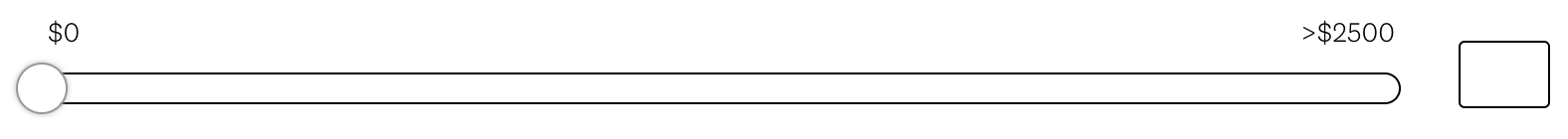


18. If you received direct payments/hazard pay, did you feel that this amount was sufficient? (Select one)

- Yes
- No
- N/A
- Other (specify)

19. Which of the following psychiatric/psychological services were offered by your institution and which did you find helpful? Rate the effectiveness on a scale of 1 (not at all effective) to 5 (very effective). Select N/A if not offered.

| Services | 1 | 2 | 3 | 4 | 5 | N/A |
| --- | --- | --- | --- | --- | --- | --- |
| One-on-one in-person therapy | Ο | Ο | Ο | Ο | Ο | Ο |
| One-on-one virtual therapy (phone or video call) | Ο | Ο | Ο | Ο | Ο | Ο |
| Group in-person therapy | Ο | Ο | Ο | Ο | Ο | Ο |
| Group virtual therapy (phone or video call) | Ο | Ο | Ο | Ο | Ο | Ο |
| Other (specify) | Ο | Ο | Ο | Ο | Ο | Ο |

20. Were there any out-of-pocket fees you needed to pay for institution-sponsored psychiatric/psychological services? (Select one)

- Yes
- No
- N/A
- Other (specify)

21. At any point, did you feel obligated to contact the psychiatric/psychological services offered by your institution? (Select one)

- Yes
- No
- N/A
- Other (specify)

Page 3: Suggestions for the Future

22. Are there any specific wellness initiatives which you wish had been offered?

Enter text

23. What else may have improved morale?

Enter text

24. Anything else that you wish to tell us?

Enter text

**Appendix C: Membership Numbers (Medical Societies and Facebook Groups)**

**Table A1:** Medical society listserv and closed Facebook group membership numbers, as measured at the time of survey distribution on these platforms between July 25^th^ and August 8^th^, 2020. As these numbers are dynamic and change day-to-day, membership was noted in the middle of the active data collection period (8/1/2020) and rounded to the nearest 100 members.

| **Platform** | **Membership** |
| --- | --- |
| ACEP | - |
| All Members | 41,800 |
| CORD | - |
| Survey Community | 2,600 |
| SAEM | - |
| Educational Research Interest Group | 600 |
| RAMS | 4,100 |
| Facebook Groups |  |
| EMDocs | 22,700 |
| Emergency Physician Forum | 4,300 |
| **Total** | **76,100** |

**Appendix D: STROBE Checklist**

*All of the following items were addressed in the manuscript submission for the present study.*

Title and abstract

- Indicate the study’s design with a commonly used term in the title or the abstract
- Provide in the abstract an informative and balanced summary of what was done and what was found

Introduction

- Background and rationale: Explain the scientific background and rationale for the investigation being reported
- Objectives: State specific objectives, including any prespecified hypotheses

Methods

- Study design: Present key elements of study design early in the paper
- Setting: Describe the setting, locations, and relevant dates, including periods of recruitment, exposure, follow-up, and data collection
- Subjects: Give the eligibility criteria, and the sources and methods of selection of subjects
- Variables: Clearly define all outcomes, exposures, predictors, potential confounders, and effect modifiers. Give diagnostic criteria, if applicable
- Data sources/measurement: For each variable of interest, give sources of data and details of methods of assessment (measurement). Describe comparability of assessment methods if there is more than one group
- Bias: Describe any efforts to address potential sources of bias
- Study size: Explain how the study size was arrived at
- Quantitative variables: Explain how quantitative variables were handled in the analyses. If applicable, describe which groupings were chosen and why
- Statistical methods:
  - Describe all statistical methods, including those used to control for confounding
  - Describe any methods used to examine subgroups and interactions
  - Explain how missing data were addressed
  - If applicable, describe analytical methods taking account of sampling strategy
  - Describe any sensitivity analyses

Results

- Participants
- Report numbers of individuals at each stage of study, e.g., numbers potentially eligible, examined for eligibility, confirmed eligible, included in the study, completing follow-up, and analyzed
- Give reasons for non-participation at each stage
- Consider use of a flow diagram
- Descriptive data
- Give characteristics of study subjects (e.g., demographic, clinical, social) and information on exposures and potential confounders
- Indicate number of subjects with missing data for each variable of interest
- Outcome data: Report numbers of outcome events or summary measures
- Main results
  - Give unadjusted estimates and, if applicable, confounder-adjusted estimates and their precision (e.g., 95% confidence interval). Make clear which confounders were adjusted for and why they were included
  - Report category boundaries when continuous variables were categorized
  - If relevant, consider translating estimates of relative risk into absolute risk for a meaningful time period
- Other analyses: Report other analyses done, e.g., analyses of subgroups and interactions, and sensitivity analyses

Discussion

- Key results: Summarize key results with reference to study objectives
- Limitations: Discuss limitations of the study, taking into account sources of potential bias or imprecision. Discuss both direction and magnitude of any potential bias
- Interpretation: Give a cautious overall interpretation of results considering objectives, limitations, multiplicity of analyses, results from similar studies, and other relevant evidence
- Generalizability: Discuss the generalizability (external validity) of the study results

Other information

- Funding: Give the source of funding and the role of the funders for the present study and, if applicable, for the original study on which the present article is based
